# Laparoscopic Incidental Finding of Pneumatosis Intestinalis in Acute Appendicitis

**DOI:** 10.1155/2014/709874

**Published:** 2014-08-21

**Authors:** N. Mayooran, A. Olu Shola, N. Iqbal

**Affiliations:** ^1^Sligo General Hospital, Sligo, Ireland; ^2^Maidstone Hospital NHS Trust, Kent, UK; ^3^Letterkenny General Hospital, Donegal, Ireland

## Abstract

Pneumatosis intestinalis (PI) is a rare condition where the gas trapped inside the bowel wall. It is commonly found as an incidental finding on routine abdominal imaging or scans. We present a case of incidental laparoscopic finding of pneumatosis intestinalis on a 32-year-old male, who underwent a laparoscopic appendectomy for an acute appendicitis. Laparoscopic appendectomy was performed and pneumatosis intestinalis managed conservatively. Patient did well and was discharged home. Management of PI depends on clinical presentation; asymptomatic PI can be managed adequately by treating underlying causes. We report a case of incidental laparoscopic finding of Pneumatosis intestinalis, which was adequately managed by treating underlying appendicitis.

## 1. Introduction

Pneumatosis intestinalis (PI) is an uncommon condition where the air trapped inside the bowel wall. It is usually found as an incidental finding on routine abdominal imaging. PI can be cystic, linear, and microvesicular in appearance. Mostly it is recognized as a benign condition and treated conservatively [1]. We report a case of a patient with acute appendicitis, where pneumatosis intestinalis was incidentally found on laparoscopy and managed by treating the underlying disease.

## 2. Case Report

A 32-year-old Caucasian male presented with 1 day history of right lower quadrant pain, without any bowel or urinary symptoms; vital signs were stable. On physical examination abdomen showed rebound tenderness and guarding in right iliac fossa. He recently had normal investigation for a nonspecific chest pain. He takes no regular medication and no known medical allergies. He has a significant family history for hyperlipidemia and cardiac problems. He is a nonsmoker and consumes alcohol occasionally. His white cell count was 14.2 × 10^9^/L with absolute neutrophils 12.1 × 10^9^/L, CRP 38 mg/L, and Hb 14.1 g/dL. All other blood tests were under normal limits. His urine dipstick showed no signs of infection or blood.

**Figure 1:**
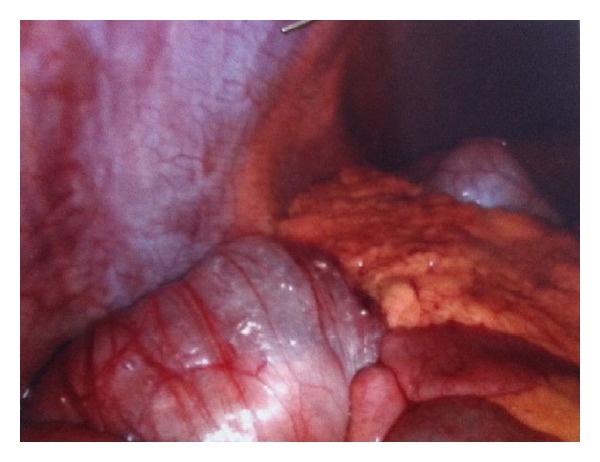
Figure shows the anterior abdominal wall above with pneumatosis intestinalis on the ascending colon with omentum and small bowel loops medially.

**Figure 2:**
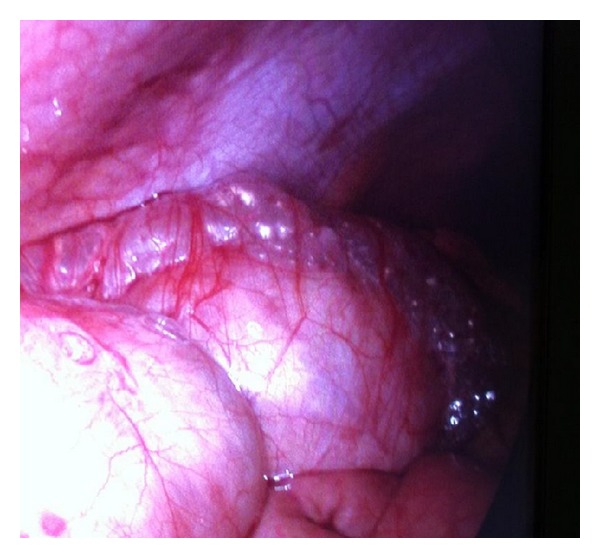
Figure shows a closure view of cystic appearance of the pneumatosis intestinalis on ascending colon with anterior abdominal wall above.

The presentation, clinical and bio chemical work up suggested acute appendicitis. Patient underwent a laparoscopic appendectomy. During laparoscopy we noted cystic gaseous changes of serosa of the ascending colon up to the hepatic flexure (Figure 1) with acutely inflamed appendix without perforation and no pus or fluids noted inside the abdomen or pelvis (Figure 2). These cystic changes revealed as of the pneumatosis intestinalis. There were no signs of perforation or ischemic bowel noted and no other intra abdominal abnormalities seen. After a good diagnostic laparoscopy, appendectomy was performed. Clinical signs and symptoms were improved after appendectomy. Patient recovered well and discharged home on 3rd post operative day for GP follow up and discharged home.

## 3. Discussion 

The appearance of gas under the subserosal layers of the bowel wall was generally termed as pneumatosis intestinalis. It was initially recognized and reported in a French literature by Du Vernoi on 1754. Since then, there were several pseudonyms used to describe it.

The historical classification divides pneumatosis intestinalis into primary (15%), which are benign cystic appearance in submucosal or subserosal layers from unknown origin [2]. The secondary (85%) are caused by obstructive pulmonary disease or with life threatening bowel conditions such as necrotic bowel or with bowel obstruction [3].

The pathophysiology of PI is not well understood. The recent hypothesis suggests that mucosal integrity, intraluminal pressure, bacterial flora, and intraluminal gas have an interactive role in the formation of the pneumocysts [4].

In our patient, although we used a non-traumatic port introducer; our initial suspicion made us look for any iatrogenic injury to the bowel wall. Fortunately there was no obvious bowel injury noted on laparoscopy. It is important to note that the initial laparoscopy was performed via umbilical port, away from ascending colon and remaining two ports were inserted under laparoscopic guidance. There can be two possible explanations for this case scenario. Firstly the inflammatory changes in appendix lead to increased mucosal perfusion and secondly increased intra-abdominal pressure due to CO_2_ inflation was similar mechanism to chronic pulmonary diseases induced PI.

Incidental findings of PI on radiology or endoscopy are normally asymptomatic and behave in a benign pattern [5]. Treating the underlying pathology is commonly adequate for this incidental PI.

PIs rarely cause abnormal clinical signs, but most commonly they do express the features of underlying pathologies. A 919 cases review study on PI published in 1979 explains that the PI in small bowel predominantly causes vomiting, abdominal distension, and diarrhoea [6, 7]. The involvement of colon can cause diarrhoea, haematochezia, abdominal pain, and distension.

Radiological investigations from plain film abdomen to CT scan can easily detect PI. Various other studies such as barium contrast study or MRI also can be used [8]. The line of management of radiological diagnosis of PI is totally depending on clinical context of the patient.

Patients with acute abdominal signs and symptoms with radiological suggestion of PI should be given additional care to look for underlying abdominal catastrophe. PI is a sign not a disease; it could represent from benign to life threatening conditions of the gastro intestinal system.

There is only handful of cases reported on PI associated with acute appendicitis. There is a case reported; PI was noted on CT scan in a patient with acute appendicitis. This led to the suspicion of small bowel ischemia. An emergency laparotomy was performed, which revealed an acute appendicitis without any ischemic signs of the small bowel. PI was managed conservatively after appendectomy [9].

Incidental finding of PI during emergency laparoscopic appendectomy creates a challenging situation for the operating surgeon. A thorough examination of intra-abdominal organs and a good clinical judgement is necessary to manage these patients.

Treatment options vary, from initiation of antibiotics therapy, oxygen inhalation, elemental diet, hyperbaric oxygen, and surgery.

Pneumatosis intestinalis can be found as an incidental laparoscopic finding in acute appendicitis; after diagnostic laparoscopy, it can be managed exclusively by performing appendectomy. Laparoscopic incidental finding of pneumatosis intestinalis does not always indicate a life threatening bowel pathology and can be managed conservatively by treating underlying causes.
